# Development of the SCNS-TARGET: a new tool to assess unmet needs in cancer patients utilising immuno-, biological or precision therapies

**DOI:** 10.1007/s11764-025-01787-4

**Published:** 2025-04-10

**Authors:** Lisa Crowe, Morven C. Brown, Jan Lecouturier, Alastair Greystoke, Desy Nuryunarsih, Ben Rimmer, Andy Bojke, Rona Bojke, James Richardson, Mary Wells, Ezinne Ezeala, Louise Carter, Adam Todd, Linda Sharp

**Affiliations:** 1 Population Health Sciences Institute, Newcastle University, Ridley 1 Building, Claremont Road, Newcastle Upon Tyne, NE1 7RU UK; 2 Newcastle University Centre for Cancer, Newcastle University, Newcastle Upon Tyne, UK; 3 Newcastle upon Tyne Hospitals NHS Foundation Trust, Newcastle Upon Tyne, UK; 4 PPI Contributors, Newcastle Upon Tyne, UK; 5 Nursing Directorate, Imperial College Healthcare NHS Trust, London, UK; 6 Department of Surgery and Cancer, Imperial College London, London, UK; 7 Department of Pharmacy, North Tyneside Hospital, Northumbria Healthcare NHS Foundation Trust, Manchester , North Shields UK; 8 Division of Cancer Sciences, Faculty of Biology, Medicine and Health, The University of Manchester, Manchester, North Shields UK; 9 The Christie NHS Foundation Trust, Manchester, UK; 10 School of Pharmacy, Newcastle University, Newcastle Upon Tyne, UK

**Keywords:** Unmet needs, Advanced cancer, Immunotherapy, Biological therapies

## Abstract

**Purpose:**

Develop an instrument to assess unmet needs in cancer patients using immuno-, biological and precision (IBP) therapies.

**Methods:**

Development followed COSMIN guidance. Instruments to assess unmet needs of advanced cancer patients were identified, and quality and content were evaluated in a systematic review (Phase 1). Semi-structured interviews with patients utilising IBP therapies (*n* = 31) and healthcare professionals (*n* = 22) explored supportive care needs (Phase 2). Phase 3 selected a base instrument to adapt, generated new items and iteratively refined these through six meetings involving professionals (*n* = 8) and public and patient involvement representatives (*n* = 9) and patient cognitive interviews (*n* = 16). Phase 4 piloted the new instrument (*n* = 50 patients).

**Results:**

Twenty-four instruments were identified; none was developed for patients utilising IBP therapies (Phase 1). Ten domains of unmet needs were identified from the interview data (Phase 2). SCNS-SF34 was selected as the base instrument. Informed by interview data, an “add-on module” (SCNS-TARGET) was developed for patients utilising IBP therapies comprising 25 questions (psychological domain, 7 items; information, 6; healthcare, 5; economic, 3; role, 2; physical, 1; social, 1; Phase 3). Levels of missingness were low; reliability varied across questions, and, on average, patients reported 7.40 (standard deviation = 8.43) unmet needs on SCNS-TARGET (Phase 4).

**Conclusions:**

SCNS-TARGET is designed for use alongside SCNS-SF34 to assess unmet needs in those using IBP therapies. Content and face validity have been established.

**Implications for Cancer Survivors:**

SCNS-TARGET can help researchers and healthcare professionals determine unmet needs and inform requirements for new services and interventions, among patients using IBP therapies.

**Supplementary Information:**

The online version contains supplementary material available at https://doi.org/10.1007/s11764-025-01787-4.

## Introduction

The number of people living with cancer is growing steadily; in 2022, for example, an estimated 53.5 million people worldwide were alive within 5 years of a cancer diagnosis, compared to 28.8 million in 2008 [1, 2]. This rise is in part due to advances in early detection, diagnosis and treatment of cancer. Since the early 2000s, there have been major advancements in the development of targeted systemic treatments—which either help the immune system recognise and attack cancer cells or attack the cancer directly—for people with advanced or metastatic cancers [3, 4]. These immuno-, biological and precision therapies (IBP) have transformed survival prospects [5], and patients with advanced disease often now live for years instead of months [6, 7].

These innovations mean that there is an increasing demand for supportive care [8], i.e. care that is given to improve the quality-of-life of people who have an illness or disease [9]. Supportive care needs arise when those living with cancer require help with adjustment or managing the consequences of the disease and its treatment. These needs can be diverse and relate to medical or non-medical matters such as physical after-effects, psychological or economic consequences or social or spiritual concerns resulting from having been diagnosed with cancer [10–12]. However, there is considerable evidence, from different settings, time periods and healthcare systems that many of these needs go unmet. For example, a 2022 systematic review conducted by Hart et al. [13] concluded that between one quarter and one half of those living with advanced cancer experience unmet needs related to finances, the health system or information, psychological issues and physical and daily living.

To inform the development and provision of cancer supportive care services or interventions, it is important to be able to determine the nature and frequency of unmet needs. There have been many instruments developed to assess unmet needs in cancer patients, and there is extensive heterogeneity in their development, content and quality [14]. While the utility of these instruments has been well characterised (see, for example, [15–17]), they were largely developed before the advent—and widespread use—of IBP therapies. As well as being new, there are some fundamental differences between how these drugs are utilised compared to traditional cytotoxic chemotherapies: modes of administration, treatment monitoring requirements and treatment durations differ. Chemotherapy courses are often short and time-limited while, in contrast, patients may utilise an IBP therapy for years; consequently, any resulting toxicities tend to be acute and periodic with chemotherapy but could be chronic with an IBP therapy. The specific toxicities experienced also differ. For example, in lung cancer, the chemotherapies pemetrexed and carboplatin are delivered in 4–6 cycles of 3 weeks, with common side-effects including risk of infection, flushing, shivering, itching or feeling dizzy or sick [18]. In contrast, the immunotherapy pembrolizumab is given for at least 2 years; immune-related side-effects are common, and patients’ kidney and liver function need to be carefully monitored [19].

These differences, in turn, have the potential to impact on patients’ experiences of care and influence their supportive care needs. For example, research suggests that oncology teams shape patients’ expectations of IBP therapies resulting in patients feeling very hopeful and positive [20]. However, patients who do not have a long-term response can feel overwhelming disappointment; hence, some may need support with managing such intense, changing, emotions. In addition, the newness of the drugs can mean that some healthcare professionals are unfamiliar with them, their use and side-effects profile [21], potentially leaving patients with very specific unmet information needs, or needs for support around specific toxicities. Existing instruments may not, therefore, fully account for the unmet needs of cancer patients using the newer IBP therapies. This paper describes the steps in the development of a new instrument to assess unmet needs in cancer patients utilising IBP therapies.

## Methods

Given that this research area is well characterised and there have been many instruments previously developed to assess supportive care needs in cancer, the approach to this work was to build on current knowledge and understanding and seek to adapt an existing supportive care needs instrument or develop an additional module to use alongside an existing instrument. To achieve this, a four-phase multi-methods approach was used [22], which followed the COSMIN (COnsensus-based Standards for the selection of health status Measurement INstruments) guidance for developing patient-reported outcome measures [23–25] (Table 1). In Phase 1, we sought to identify and evaluate existing instruments to measure unmet needs in patients with advanced cancer, while Phase 2 focused on concept elicitation. These findings were used to inform Phase 3 in which the new instrument was developed. Phase 4 involved pilot testing of the instrument. Supplementary Table 1 includes a completed COSMIN study design checklist for the current study.

**Table 1:** Summary of the phases and activities in the development of SCNS-TARGET

Purpose	Activity	Outcome
Phase 1: Identification and evaluation of existing instruments		
To identify available instruments to assess unmet needs of advanced cancer patients	Systematic review assessing the development, content and quality of available instruments to assess unmet needs	A list of 24 instruments of variable quality which formed a "pool" from which to select one for possible adaptation Identification of 11 domains of unmet need which may be present in advanced cancer patients
Phase 2: Concept elicitation		
To identify supportive care needs experienced by patients using IBP therapies	Qualitative interviews with 31 cancer patients receiving various IBP therapies Qualitative interviews with 22 healthcare professionals involved in the care of patients receiving IBP therapies	Summary of unmet needs for patients receiving IBP therapies, mapped to framework of 11 domains of unmet need
Phase 3: Instrument development		
To develop the draft instrument	Selection of a base instrument (from Phase 1) Development of a module to add to the base instrument, consisting of: • Reviewing unmet need domains (from Phase 2) • Generating items • Refining items through workshops, cognitive interviews and patient and public involvement (PPI)	Draft instrument developed and refined
Phase 4: Pilot and finalise		
To finalise the new instrument	Draft instrument piloted with 50 cancer patients in receipt of IBP therapies; participants invited to complete the instrument again after a 2-week interval to determine test–retest reliability	SCNS-TARGET finalised, comprising 25 item add-on module to SCNS-SF34 for those on IBP therapies

Our focus on targeted systemic therapies encompassed (i) immunotherapies (i.e. medicines which stimulate or restore the ability of the immune system to detect and destroy cancer cells), (ii) biological therapies (i.e. monoclonal antibodies that block key pathways for cancer growth or survival) and (iii) precision therapies (i.e. treatments tailored to account for genomic variations in the tumour). Although endocrine therapies may also be considered targeted, these were excluded from consideration as patient experiences of these drugs have been extensively examined.

### Phase 1: Identify and evaluate existing instruments

A systematic review was undertaken to identify and evaluate content and quality of available instruments to assess unmet needs of advanced cancer patients. Full details have been reported elsewhere [26]. In brief, searches of MEDLINE, CINAHL, Embase, PubMed and PsycINFO were performed from database inception to 11 January 2021. Data were abstracted on characteristics, development and content of eligible instruments. Instrument items were classified according to 11 possible unmet needs domains, namely, activities of daily living, autonomy, economic, healthcare, information, physical, psychological, role, social, sexuality and spiritual (definitions provided in Supplementary Table 2). These domains were developed by scrutinising, then categorising by topic, each relevant item in the eligible instruments. Quality appraisal followed COSMIN guidelines (23) and focused on methodological and quality assessment, GRADE, feasibility and interpretability.

### Phase 2: Concept elicitation

Phase 2 sought to identify supportive care needs experienced by patients utilising IBP therapies. Full methods and findings are reported elsewhere [27]. In brief, qualitative semi-structured interviews were conducted between June 2021 and April 2022 by either LC (social scientist) or MCB (health psychologist), both experienced qualitative researchers who had had no communication or relationship with participants prior to the study. Interviews were undertaken with patients with advanced cancer who had experience of using IBP therapies. Eligible cancers included lung, colorectal, ovary, (female) breast, renal or malignant melanoma. Patients could have previously received other cancer treatments (e.g. chemotherapy), but a period of at least 3 months had to have passed to ensure the data captured related to the unmet needs associated with receiving IBP therapies. Breast cancer patients receiving endocrine therapies alone were not eligible. Patients were selected for maximum variation using a purposive sampling strategy focusing on cancer type and gender. Health professionals involved in providing hospital care for patients receiving IBP therapies from any of the tumour groups were also interviewed. Interviews explored experiences of IBP and support needs and were audio-recorded, transcribed verbatim and anonymised. Separate topic guides were developed for each interview set; these were used flexibly, with participants free to discuss other issues. Data were analysed using the framework approach [28] with the framework comprising the 11 potential domains of unmet need (Supplementary Table 2). Initially, five interview transcripts were independently and inductively coded by LC and MCB. After agreeing the “fit” of the framework, the remaining interviews were deductively coded, with 50% of the transcripts being double coded. Interviews were undertaken until data saturation was reached (assumed when subsequent interviews did not lead to the identification of any "new" unmet needs). Patient interviewees were asked whether they would be willing to participate in later phases of the study.

### Phase 3: Instrument development

Phase 3 comprised three steps: selection of a base instrument to adapt/augment, generation of items and refinement of these items.

#### Step 1

To select and define requirements for the base instrument, the research team reviewed those identified in Phase 1. Instruments were excluded if they were (i) not targeted specifically to people with cancer, (ii) population-specific (e.g. designed for a specific ethnic/minority group), (iii) intended for completion in a specific setting (e.g. Emergency Department) or (iv) not designed for self-completion. Others were excluded where the English version had not been validated or the patient burden (length, time to complete) was considered excessive (e.g. time to complete more than 15 min). Where any of this information was unclear, corresponding authors of relevant papers were contacted to seek clarification. The remaining candidate instruments were discussed at a workshop with a cancer public and patient involvement (PPI) group who provided opinions on the instrument layout, length, question format and response options. Following this, and after seeking clarification around access and copyright issues, a base instrument was selected. After comparison of Phase 2 findings with the content of the base instrument, it was decided to develop an add-on module.

#### Step 2

In a series of meetings, the research team systematically reviewed the findings from Phase 2 domain by domain, considering specific needs identified from the interviews against base instrument content. If the need was not already included, an item for the add-on module was drafted; if the need was already captured, nothing was added. In drafting items, new items were worded to be consistent, as far as possible, with the item format used in the base instrument. Cognisant of participant burden, the team sought to minimise the number of new items when possible.

#### Step 3

To ensure comprehensibility, content validity and face validity, the draft add-on module was pre-tested and refined in a series of events held from May 2022 to November 2022: (i) meetings with health professionals from all relevant disciplines (e.g. oncology, pharmacy) involved in the care of patients utilising IBP therapies and cancer, (ii) meetings with PPI representatives and (iii) three rounds of cognitive interviews with cancer patients using IBP therapies. Eligibility for cognitive interviews was the same as for Phase 2, and interviews were conducted with a mix of new participants and several people who had participated in Phase 2. Different patients participated in each round. Meetings were facilitated by LC, and cognitive interviews were conducted by LC or MCB. The module content was refined after each meeting and/or round of cognitive interviews; the updated iteration of the module was pre-tested in the subsequent round of meetings/interviews. Issues discussed at the meetings included question relevance, comprehensibility of instructions, recall period, question wording, response options and whether any pertinent issues were missing (i.e. comprehensiveness). During cognitive interviews, patients were asked to “talk aloud” while going through the module question-by-question with verbal probing to clarify any problems or issues, as well as comprehensiveness [29]. Interviews and workshops were audio-recorded and transcribed verbatim. After each round, analysis involved identifying and systematically categorising problems and areas potentially requiring change [30]. The research team discussed findings and agreed changes to the module. Once no new issues arose, the research team re-reviewed and finalised the order of questions; consideration was given to balancing a desire to bring together questions which were related (either conceptually or in terms of how issues were discussed in Phase 2 interviews) with avoiding respondent non-differentiation in ratings (“straightlining”).

### Phase 4: Pilot and finalise

The new self-administered instrument (comprising the base instrument plus the add-on module) was piloted among 50 patients receiving IBP therapies for lung, breast, renal, ovary or colorectal cancer or melanoma. Participants were recruited July–November 2022 primarily using a convenience sampling strategy across three hospitals in the National Health Service (NHS), the UK’s publicly funded healthcare system. This was supplemented with a small number of patients who had been interviewed in Phase 2 and agreed to help with later phases. The instrument was completed on paper and posted back to the research team using a self-addressed envelope.

The survey also collected data related to socio-demographic characteristics of the participants and included a free-text section where participants could share any other information with the research team. Participants were invited to complete the instrument a second time after a 2-week interval to determine test–retest reliability of the SNCS-TARGET questions, which was assessed using Cohen’s (linear) weighted kappa. A 2-week interval was chosen as patients using IBP therapies are typically reviewed clinically every 1–3 months, so it would be reasonable to expect needs would largely be stable over 2 weeks. The test conditions were similar for the two administrations (e.g. information provided to participants, how administered). In terms of measurement error, for each question, percentages of positive and agreement were also computed between the two administrations; for these calculations, and for kappa, the response options were collapsed to Yes (I need help/I no longer need help) and No. Distributions of missingness and question responses on the first administration of the survey were documented overall, and means were computed. To provide an indication of known-groups validity, mean number of unmet needs reported was compared between  patient subgroups which, based on past studies [31, 32], might be expected to differ (defined by age-group (< 65, 65 +) and sex). Free-text responses were reviewed by the research team to identify any further revisions or refinements required.

## Results

### Phase 1: Identify and evaluate existing instruments

The systematic review identified 24 instruments to assess unmet needs for patients with advanced cancer (Supplementary Table 2). Two were developed for general palliative patients; eight were intended only for advanced cancer patients, and 14 were for cancer patients of all stages (but had included those with advanced cancer in their development). No instrument was developed specifically for, or based on the needs of, patients using IBP therapies. There was extensive heterogeneity in the development, content and methodological quality of the included instruments; no instrument reported all of the COSMIN measurement properties. Instruments included items from between 5 and 11 unmet needs domains, with number of items ranging from 9 to 138.

### Phase 2: Concept elicitation

Table 2 provides details of the 31 patients interviewed. The 22 health professionals interviewed comprised oncologists (*n* = 9; 3 registrars and 6 consultants), cancer nurses (*n* = 6), pharmacists (*n* = 5) and psychologists (*n* = 2). They had experience of working with patients with lung cancer (*n* = 10 professionals), breast cancer (*n* = 9), melanoma (*n* = 8), ovarian cancer (*n* = 7), renal cancer (*n* = 5) and colorectal cancer (*n* = 4). Patients were recruited through four NHS hospital sites (*n* = 27) or cancer charities (*n* = 4). Health professionals were recruited through four NHS hospital sites (*n* = 19) or social media (*n* = 3). For the hospital site recruitment, potential participants were screened for eligibility by the local principal investigators. Interviews provided evidence of supportive care needs in 10 of the 11 unmet needs domains (the exception was spirituality); no needs were identified which did not map to the pre-specified domains. Two groups of unmet needs were identified. The first group related to utilising IBP therapies (e.g. specific treatment side effects (physical domain), living long-term with uncertainty (psychological domain), others not understanding why the person with cancer was not cured (social domain)). The second group of needs related to living with (advanced) cancer (e.g. retiring from work (role domain), loss of independence (autonomy domain), accessing information about social welfare benefits (economic domain)). Needs could occur together. They sometimes persisted long-term and, other times, first presented a considerable time after treatment commenced.

**Table 2:** Characteristics of patients who participated in Phase 2 interviews, Phase 3 cognitive interviews and first administration of Phase 4 pilot survey

Participant characteristics	Phase 2: Concept elicitation	Phase 3: Instrument development^a^	Phase 4: Pilot survey^b^
Sex			
Male	11	3	15
Female	20	13	35
Age			
31–40	2	0	2
41–50	3	4	4
51–60	5	3	12
61–70	12	1	14
Older than 70	8	2	16
Not reported	-	6	2
Cancer site			
Breast	8	6	15
Colorectal	1	0	2
Lung	8	5	13
Malignant melanoma	5	2	1
Ovary	3	1	3
Renal	6	2	14
Not reported	-	-	2
Treatment class^c^			
Monoclonal antibodies	4	2	-
Immunotherapies	1	0	-
CDK4/6 inhibitors	5	2	-
PARP inhibitors	3	0	-
Tyrosine kinase inhibitors	15	5	-
Combination	3	2	-

^a^Characteristics shown are for those who participated in cognitive interviews; 11 had previously been interviewed in Phase 2 interviews. In addition to the 16 cognitive interviews, nine PPI representatives (people with cancer) took part in meetings to discuss the instrument; their characteristics were not recorded

^b^One participant had previously participated in Phase 2 interviews

^c^Recruiting sites invited patients utilising IBP therapies to complete the pilot survey. Information on the specific treatment(s) participants were utilising was not available; the anonymous nature of the survey precluded this information being obtained from participants’ medical records

### Phase 3: Instrument development

#### Step 1

Twenty-one of the 24 instruments identified in Phase 1 were excluded following review against the requirements set for the base instrument (Supplementary Table 3). Three were shortlisted for further consideration: Cancer Support Source (CSS-25), Electronic Holistic Needs Assessment (eHNA) and SCNS-SF34 [33–36]. These were reviewed by PPI representatives. Perceived advantages and disadvantages of each are summarised in Supplementary Table 3. The SCNS-SF34 was selected because it is easily and freely available, widely used and internationally validated; it covers multiple domains and other groups have developed add-on modules.

#### Step 2

Phase 2 interview findings were compared with the items included in the SCNS-SF34. The existing SCNS-SF34 questions captured three of the 10 unmet needs domains highlighted in Phase 2 (activities of daily living, autonomy, sexuality). Items were drafted for the remaining seven domains; example items are shown in Table 3. The add-on module that went forward for pre-testing included 23 new items. Because concerns had previously been expressed about the five-level response format in the SCNS-SF34, this draft included a three-level response previously used [37] (*Stem*: In the last month did you need help with any of the following:…. *Response options*: No, I did not need help/Yes, but I no longer need help/Yes, I need help).

**Table 3:** Domains of unmet need included in base instrument (SCNS-SF34) and new module (SCNS-TARGET) and examples of new items added

Domain^a^	Items included in SCNS-SF34	Items in add-on module, SCNS-TARGET^b^	Example new items
Activities of daily living	x	-	-
Autonomy	x	-	-
Economic	-	x	• Accessing information about financial support that may be available • Accessing help with how to apply for financial support that may be available
Healthcare	x	x	• Accessing your cancer treatments (e.g. getting to the hospital, parking, receiving a delivery of medication, appointments changing/being cancelled) • Taking or having your cancer treatment
Information	x	x	• Being informed about what to do if you miss a dose of your treatment • Knowing if any symptoms you experience are caused by your cancer treatment
Physical	x	x	• Managing side-effects of treatment on a day-to-day basis
Psychological	x	x	• Worries that a dose reduction, or having to stop treatment early, will mean the treatment will not work • Worries about what will happen next if your current treatment stops working
Role	-	x	• Worries about not being able to work
Sexual	x	-	-
Social	-	x	• Accessing support about how to talk to people about your cancer

^a^Domains defined as per Phase 1 systematic review (Supplementary Table 2; 22). Spiritual domain not shown; not included in SCNS-SF34 and no spiritual unmet needs were raised in Phase 2 interviews (22)

^b^Dash indicates that domain was raised in Phase 2 interviews but that no additional items were added; items in SCNS-SF34 were considered to cover issues raised by patients on IBP therapies

#### Step 3

The draft was presented, discussed and refined through a series of four stakeholder meetings (attended by eight health professionals in total: two hospital pharmacists, two cancer nurse specialists, three oncologists, one psychologist), two cancer patient workshops (nine PPI representatives in total, eight in each workshop) and three rounds of cognitive interviews (16 patients: round 1, *n* = 5; round 2, *n* = 5; round 3, *n* = 6). Characteristics of patients who took part in cognitive interviews are shown in Table 2. This iterative pre-testing identified a range of lexical, logical, omission/inclusion and computational problems with the draft module; examples are shown in Table 4, together with information about how items were revised to address these. In terms of temporal problems, queries were raised about the recall window and whether 1 month was too short in the context of treatments which patients may be on long term. We concluded that, for consistency with the base instrument, the recall window should remain 1 month; the developers of the SCNS justified 1 month as a recent and well-defined period [38]. In terms of comprehensiveness, two further items were added after the first interview round (“accessing support about how to talk to people about your cancer”, “accessing other sources of support outside of your cancer care team and family/friends”). Health professionals suggested adding a list of side-effects to the item about needing “support knowing what side-effects to tell your doctor/nurse”. We decided that this would be unwieldy and, because such a list would inevitably be treatment specific and IBP therapies are evolving rapidly, might mean the module would not be relevant in the future. Changes after rounds two and three involved rewording and reordering, the latter so that related items were located next to one another. Concerns were raised by a few patients in workshops and interviews about the response options being potentially confusing; in particular, the need for two “yes” options was questioned. As the three-level response option had previously been published [37], it was decided to take this format forward for piloting.

**Table 4:** Examples of problems encountered in Phase 3 pre-testing and responses to these

Problem class	Description	Example of problems identified during pre-testing	Responses to example problems
Lexical	Meaning of words not known or inability to use words in question or response context	“Support using your cancer treatments as directed by doctor” Is “using” the most appropriate word, given some patients receive treatment via intravenous injection or infusion and do not necessarily use their treatment?	Changed to “Taking or receiving your cancer treatment” Added “receiving” to text to ensure people receiving treatment administered via different routes (e.g. by injection or infusion) were able to answer this question
		“Being adequately informed about why I might be offered a dose reduction” This phrase was understood by participants, but issues were raised about the relevance of the term “dose reduction” to immunotherapy treatment considering patients are not offered dose reductions with these types of therapies. In this context, immunotherapy will be stopped earlier rather than being dose reduced	Changed to “Understanding why I might be offered a dose reduction OR have to stop treatment early” The updated wording included text about stopping treatment early, which is more appropriate for people using immunotherapies
Temporal	Problem with the time period or time spent on an activity related to the question, including misunderstanding the descriptions of time used in the question	“…indicate whether you needed help with this issue in the last month…” The recall period of 1 month for patients was raised as a potential problem. For example, it was suggested that, due to the prolonged duration of some IBP treatments, a recall period of 1 month could potentially be too short for this patient group	After discussion, the recall period for the instrument remained as 1 month, as is in line with the original SCNS SF-34 instrument
		“Coping/Coming to terms with having an advanced cancer” It was questioned if using the term “coping” was appropriate, given this can change with time. Participants queried whether the question could be more specific given that coping is a broad term and could potentially have different meaning to different people	Changed to “Coming to terms with having cancer” Removing the term “coping” to make the question more specific
Logical	Problem with question wording because of “connectives” such as “and” and “or” “False presuppositions” within a question, i.e. a question is asked in such a way that it assumes something about a respondent which is not true	“Accessing help with how to apply for financial support that may be available” It was suggested that a “not applicable” box was needed given this question may not be relevant for all patients	A “not applicable” box was not added to the instrument for several reasons: - The original SCNS has does not include a “not applicable” response option - If the question is not relevant to the patient, then they have no unmet needs (so the answer “no” applies)
		“Support identifying relevant side effects” It was questioned whether “relevant” is an appropriate term for the item—given the term is subjective. It was also questioned if the term “side effects” was appropriate given some things might not be attributed as side effects by patients	Changed to “Knowing if any symptoms you experienced are caused by your cancer treatment” The terms “relevant” and “side effects” were removed to make the item less subjective. The term “knowing” was added to make the item more related to information needs
Omission/inclusion	Problems where participants are unsure of the scope which is covered by a question	The use of the term “doctors” did not appear to sufficiently describe the breadth of healthcare professionals involved in the care of patients with cancer	The term “doctors” was changed to “cancer care team” in all relevant questions
		“Taking your cancer treatments” If a patient is receiving their medication by infusion or injection, the term “taking” is not necessarily appropriate	Changed to “Taking, or having, your cancer treatment” This updated wording covered patients who were taking an oral therapy and those who were receiving treatment intravenously
Computational	All other problems, e.g. problems with long, complex questions, memory or use of mental arithmetic	“Being adequately informed about why I might be offered a treatment break” This item was deemed to be overly complex	Changed to “Understanding why you might be offered a treatment break” The updated item was simplified
		“Feeling you have lost yourself in being a cancer patient” It was questioned if the wording “lost yourself” was appropriate as some people were not entirely clear what the item was referring to	Changed to “Feeling you have lost your identity through being a cancer patient” The term “identity” was introduced to make it clear the item related to identity

### Phase 4: Pilot and finalise

The pilot tested survey included the SCNS-SF34 plus the new module comprising 25 questions for those utilising IBP therapies, named the SCNS-TARGET. Of the new questions, seven related to the psychological domain, six to information, five to healthcare, three to economic, two to role and one each to physical and social (Table 5).

**Table 5:** Summary of question responses from first administration of the Phase 4 pilot survey, SCNS-TARGET^a^

Question stem	Domain^b^	Response	
*Please indicate whether you needed help with this issue within the last month as a result of having cancer……*		Missing	Yes, I did need help
35.Accessing cancer treatments (e.g. getting to the hospital, parking, receiving a delivery of medication, appointments changing/being cancelled)	Healthcare	2 (4%)	9 (18%)
36.Support taking or receiving your cancer treatment	Healthcare	2 (4%)	3 (6%)
37.Information on what to do if you miss a dose of treatment	Information	1 (2%)	2 (4%)
38.Knowing if any symptoms you experience are caused by your cancer treatment	Information	2 (4%)	4 (8%)
39.Knowing what side effects to tell your cancer care team	Information	2 (4%)	8 (16%)
40.Talking about something that is embarrassing or sensitive	Psychological	2 (4%)	3 (6%)
41.Managing side effects on a day-to-day basis	Physical	3 (6%)	5 (10%)
42.Understanding why I might be offered a treatment break	Information	2 (4%)	6 (12%)
43.Understanding why I might be offered a dose reduction OR have to stop treatment early	Information	2 (4%)	7 (14%)
44.Worrying that a dose reduction OR having to stop treatment early will mean the treatment won’t work	Psychological	4 (8%)	11 (22%)
45.Worrying that current treatment might stop working	Psychological	2 (4%)	16 (32%)
46.Worrying about what is next if current treatment stops working	Psychological	2 (4%)	17 (34%)
47.Coming to terms with having cancer	Psychological	3 (6%)	8 (16%)
48.Learning to live with an uncertain life expectancy	Psychological	3 (6%)	12 (24%)
49.Feeling your life is dominated by your cancer	Psychological	3 (6%)	12 (24%)
50.Feeling you have lost your identity through being a cancer patient	Role	2 (4%)	9 (18%)
51.Accessing information to share with family and friends so they understand your cancer and treatment	Information	1 (2%)	6 (12%)
52.Accessing support about how to talk to people about your cancer	Social	2 (4%)	3 (6%)
53.Having the opportunity to talk about things that are important to you	Healthcare	2 (4%)	5 (10%)
54.Accessing other sources of support outside of your cancer care team and family/friends	Healthcare	2 (4%)	8 (16%)
55.Accessing information about financial support that may be available	Economic	1 (2%)	10 (20%)
56.Accessing help with how to apply for financial support that may be available	Economic	1 (2%)	12 (24%)
57.Worrying about financial security/future for myself or my family	Economic	1 (2%)	16 (32%)
58.Worrying about not being able to work	Role	1 (2%)	12 (24%)
59.Having the opportunity to talk about death and dying	Healthcare	3 (6%)	8 (16%)

^a^Questions are numbered from 35 onwards as this module is intended to be used with the SCNS-SF34, which includes 34 items

^b^Domains were allocated empirically by the research team, following the definitions within Phase 1 systematic review (Supplementary Table 2; (22))

The characteristics of the 50 patients who completed the first administration of the pilot survey are shown in Table 2. In the first administration, levels of missing data were low. One new item (“worrying that a dose reduction OR having to stop treatment early will mean the treatment won’t work”) was missed by four (8%) participants, and four questions were missed by three (6%) participants (Table 5). On average, participants did not complete 1.70 (standard deviation (sd) = 5.28) of the unmet need questions in total (SCNS-SF34: mean = 0.68, sd = 1.93; SCNS-TARGET: mean = 1.02, sd = 4.01); this did not vary significantly by sex or age-group (data not shown).

Across SCNS-TARGET items, between 4 and 34% of patients reported that they had an unmet need for help (Table 5). The most frequently reported unmet needs, endorsed by a third of participants, were psychological (“worrying that the treatment might stop working”, 32%; “worrying about what is next if current treatment stops working”, 34%) and economic (“worrying about financial security/future for myself or my family”, 32%). On average, across both SCNS elements, participants reported 10.14 (sd = 12.64; range 0 to 50) unmet needs (SCNS-SF34: mean = 5.90, sd = 7.43, range 0 to 29; SCNS-TARGET: mean = 4.24, sd = 5.68, range 0 to 21). The mean number of unmet needs on the SCNS-TARGET was higher among younger than older participants (< 65 years: 7.40 (sd = 8.43); 65 + years: 1.86 (sd = 3.56); *p* = 0.002) but varied little by sex (males: 3.53 (sd = 5.50); females: 4.52 (sd = 5.81); *p* = 0.563).

Among the 50 participants, 27 completed the second administration. For individual SCNS-TARGET questions, reliability varied; it was in the range of fair or less (≤ 0.40) for 8 questions, moderate (0.41–0.60) for 11 and at least substantial (≥ 61) for 6. Positive agreement ranged from 0.82 to 1 across questions (median 0.94) and negative agreement from 0 to 1 (median 0.53).

In terms of free-text responses, one participant suggested that questions be added on reproductive concerns; we did not action this as it had not been raised by participants in Phase 2 or 3. It was suggested that the response options be simplified; these were revised to “No, I did not need help/Yes, I did need help”. No other changes were made.

## Discussion

The purpose of this study was to develop an instrument to measure unmet supportive care needs in cancer patients utilising IBP therapies. The four phases have resulted in the SCNS-TARGET, a new module of 25 questions designed to use alongside the SCNS-SF34 in patients utilising these treatments. The new module has both face and content validity. Levels of missing data were low. In terms of known-groups differences, compared to older patients, younger patients had more unmet needs on the SCNS-TARGET. While this finding should be interpreted with caution given the sample size, the pattern is broadly consistent with studies in other cancer patient populations [31, 32].

The addition of 25 questions to the SCNS-SF34 supports the proposition that the unmet needs of patients on these new treatments may not be entirely the same as those experienced by patients on traditional cytotoxic drugs. In our Phase 1 systematic review [26], we reviewed items in each instrument identified and classified these into one of 11 domains; SCNS-SF34 items were categorised as falling into seven domains. The questions in the SCNS-TARGET also encompassed seven domains, four of which overlap with the SCNS-SF34 domains (physical, psychological, information, healthcare). Seven, six and five new questions, respectively, were developed relating to psychological, information and healthcare-related unmet needs. The growing qualitative evidence-base on the lived experience of patients on these treatments highlights psychological issues (such as challenges around living with uncertainty) and the opportunities for improving healthcare experiences [7, 39–41]. These findings are broadly consistent with the items included in SCNS-TARGET. What was striking here was how many different unmet information needs patients experienced. Perhaps unsurprisingly, several of the new items on information (such as those relating to understanding why a treatment break might be offered or worries about the treatment stopping working) reflect differences in how these new drugs are used compared to traditional chemotherapy. This suggests that health systems may need to improve information provision for patients on IBP therapies. Co-design approaches, involving working in partnership with patients to develop appropriate and accessible information, may be a useful way forward [42].

The SCNS-TARGET also includes questions in three domains that are not included in the SCNS-SF34: social (*n* = 1 question), role (*n* = 2 questions) and economic (*n* = 3 questions). Regarding role, the items added related to (i) loss of identity and (ii) worrying about not being able to work, which, in the Phase 2 qualitative work, resulted in concerns not only about lost income, but also about loss of purpose in life [27]. Others have noted that, as those with metastatic cancer are now living longer, it is important to pay greater attention to patients’ desires and capacities to maintain employment [43]; the SCNS-TARGET provides a potential means to undertake an assessment of whether support around work participation or resumption is desired.

In terms of the new items in the economic domain, it is well recognised that IBP therapies are costly for health services [44, 45]. Studies are emerging suggesting that utilisation of these medications can result in financial toxicity for patients [46, 47]. The recent ESMO consensus statement on screening and management of financial toxicity in cancer recognises that such risks are present both in settings where insurance coverage is absent or partial (and co-payments are required) and in publicly funded health systems, when agents are prescribed for off-label indications and may not be covered [48]. The current research was conducted in England where all residents are entitled to cancer care from the NHS free at the point of delivery and where the use of these drugs is almost entirely (if not entirely) within the NHS; thus, patients do not have to make out-of-pocket payments, or co-payments, for the drugs. The questions added in this domain relate, therefore, to non-treatment-related financial support that may be available (e.g. social welfare benefits) and worries about future financial security. The implication of this is that, if used in other settings where patients need to contribute financially for care, it may be important to capture more detail around economic unmet needs related to the costs of the medications.

Although the team sought, as far as possible, to follow the format of the base instrument selected for adaptation (i.e. SCNS-SF34), the response options in the original 5-level format—ranging from “no need” (score 1) to “high need for help” (score 5)—[33] were modified. Past studies have noted that patients experience difficulty with these options [49]. In light of this, an alternative 3-level response options, which had been previously used in a survey of head and neck cancer survivors in Ireland [37], was originally tested. However, in workshops, cognitive interviews and the pilot study, some patients found these three options confusing. The response options were therefore simplified, at the end of this development work, to two levels for both the SCNS-SF34 and SCNS-TARGET indicating whether the respondent had (score 1) or had not (score 0) needed help. Scoring of the SCNS-SF34 using the original response options is straightforward (compute Likert summated scale by summing the individual items within each subscale; this can then be standardised) [50]. While we have yet to establish the factor structure of the combined instrument, we would anticipate that the same simple approach to scoring would be appropriate with the new response options. As regards other instrument feasibility issues, while we did not formally assess how long it takes to complete the combined SCNS-SF34 and SCNS-TARGET, the SCNS-SF34 has an estimated completion time of 10 min [50], suggesting the combined instrument can be completed within 20 min. The SCNS-SF34 is freely available for researchers to use [50], and our intention is to make the SCNS-TARGET similarly available.

### Strengths and limitations

A major strength of this work is that the COSMIN guidance [23] informed the development process; we adhered to the recommended stages of development and validation and the numbers and types of participants at different stages. In terms of weaknesses, while the work benefited from the inclusion of the perspectives of patients and HCPs, we did not have the resources to interview informal caregivers—who may have informative views on patients’ needs—in Phase 2. Moreover, recruitment to Phase 2 occurred during the COVID-19 pandemic, and it is possible that this impacted the specific unmet needs experienced. In particular, the minimisation of in-person contact between healthcare professionals and patients to reduce risks of virus transmission may explain why so many unmet needs related to information and healthcare were identified. Patients with one of six solid tumours were included; while it is possible that unmet needs may differ in patients with other cancers, the included patients used a spread of IBP therapies, many of which are now available for patients with cancers at other sites. There was minimal ethnic variation among patient participants, despite efforts to recruit underserved populations. It is plausible that minority ethnic populations may have specific unmet needs, but empirical data appears largely limited to studies of unmet needs in selected populations (see, for example, [51–53]). However, it should not be assumed that the SCNS-TARGET will fully capture the needs in patients from (all) minority backgrounds utilising IBP therapies in all settings. In addition, few of the included patients were utilising immunotherapies alone. It is therefore important that future work evaluates the module among people of different ethnicities, those using a variety of different IBP therapies in different ways (e.g. alone, in combination, alongside other types of anti-cancer treatments), those with haematological cancers and, as alluded to earlier, in different healthcare systems.

In terms of instrument properties, the rigour of the development process, and engagement with patients and health professionals throughout, provides confidence around content and face validity. The Phase 4 pilot survey included 50 participants, in line with COSMIN recommendations [23]. Levels of missing data were low, but the reliability of the questions varied. While it is possible that new unmet needs could have developed over a 2-week period (or existing needs could have been met), this seems unlikely, given patients receiving IBP therapies are typically reviewed clinically every 1–3 months. This could also reflect the small sample size in the second administration (*n* = 27) of the instrument; one respondent changing their response from “No” to “Yes”, for example, could have a proportionately large impact. Further examination of reliability in a larger population would be valuable.

Psychometric properties of the SCNS-SF34 have been extensively reported (see, for example, [54, 55]), but we judged that a sample of 50 participants was too small to undertake full psychometric validation of the SCNS-TARGET. This means that, for example, while each new item was allocated to a domain, these domains do not necessarily act as subscales, nor does the SCNS-TARGET necessarily operate as a scale. The final phase of this work involves a survey of more than 400 patients utilising IBP therapies; the data gathered will be used to further investigate psychometric properties including structural validity (e.g. presence of subscales and, related to this, their internal consistency) and convergent and discriminant validity.

### Implications for research and practice

The primary intention in this work was to develop an instrument that could be used within a research context to assess the prevalence of unmet needs in the patient *population* utilising IBP therapies and determine which subgroups have more unmet needs. Such findings, in turn, could be used to inform the provision and targeting of interventions or services to meet those needs. However, it is also possible that the instrument could be used in a clinical context to identify the needs experienced by an *individual* patient as a prelude to providing tailored support. The recently published MASCC-ASCO Standards and Practice Recommendations set out standards for optimal survivorship care of people with advanced or metastatic cancer [56]. The new combined instrument may have considerable utility for addressing some of the priorities within this document; for example, standard 4 (Evaluated and Communicated Care) emphasises the importance of delivering “routine and systematic evaluation and monitoring of supportive care needs”.

In terms of future research, studies have explored the value of using symptom monitoring or other patient-reported outcome measures to stimulate discussions and/or support around issues important to an individual patient but which may not otherwise be covered in routine care or follow-up [57–59]. Unmet needs instruments could similarly be used in either primary care or specialised hospital-based follow-up. Research to explore whether this helps ensure patients’ needs are addressed and/or improves their experiences of care or quality-of-life would be of value.

This work has, so far, considered those using IBP for advanced cancer as a somewhat homogeneous group; in reality, this population includes patients with different underlying diagnoses and variable prognoses and—hence—potentially different unmet needs. This is worthy of further investigation. Finally, since the TARGET study commenced, IBP therapies have begun to be approved in the early disease setting (see, for example, [60]). Here, we focussed entirely on patients with advanced disease and future work to determine the applicability and utility of the SCNS-TARGET (used alongside the SCNS-SF34) in these emerging patient populations would be valuable.

### Conclusions

A rigorous development process has resulted in the SCNS-TARGET, a new instrument designed to be used alongside the SCNS-SF34, to comprehensively measure supportive care needs in cancer patients utilising IBP therapies. Content and face validity have been demonstrated. Work to establish further psychometric properties should be a priority.

## Supplementary Information

Below is the link to the electronic supplementary material.

Supplementary file1 (DOCX 53 KB)

## Data Availability

No datasets were generated or analysed during the current study.
