# Supplementary material for: Development of the SCNS-TARGET: a new tool to assess unmet needs in cancer patients utilising immuno-, biological or precision therapies
Source: J Cancer Surviv. 2025 Apr 10;20(Suppl 1):126–40. doi: 10.1007/s11764-025-01787-4 (PMC13676634; doi:10.1007/s11764-025-01787-4)
Supplement: Supplementary file 1 [file 11764_2025_1787_MOESM1_ESM.docx]

**SUPPLEMENTARY TABLE 1: Shortened COSMIN study design checklist for SCNS-TARGET: the ratings for the development work, as assessed by the research team, are shown in bold font**

| **Recommendation** | **Very good** | **Adequate** | **Doubtful** | **Inadequate** | **Where reported** |
| --- | --- | --- | --- | --- | --- |
| **General recommendations for the design of a study on measurement properties** | | | | | |
| ***Research aim*** | | | | | |
| **1.** Provide a clear research aim, including (1) the name and version of the PROM, (2) the target population, and (3) the measurement properties of interest. | **Research aim clearly described.** |  |  | Research aim not clearly described. | Introduction, Paragraph 4 AND Discussion, Paragraph 1 |
| ***Patient-Reported Outcome Measure (PROM)*** | | | | | |
| **2.** Provide a clear description of the construct to be measured. | **Construct clearly described.** |  |  | Construct not clearly described. | Introduction, Paragraph 2 |
| **3.** Provide a clear description of the development process of the PROM, including a description of the target population for which the PROM was developed. | **Development process clearly described.** |  | Development process not clearly described. |  | Methods, Phase 1-4 |
| **4.** The origin of the construct should be clear: provide a theory, conceptual framework (i.e. reflective or formative model) or disease model used or clear rationale to define the construct to be measured. | **Origin of the construct clear.** |  | Origin of the construct not clear. |  | Introduction, Paragraph 2 |
| **5.** Provide a clear description of the structure of the PROM (i.e. the number of items and subscales included in the PROM, instructions given and response options) and its scoring algorithm. | **Structure and scoring algorithm clearly described.** |  |  | Structure and scoring algorithm not clearly described. | Results, Phase 4, 25 items, 7 subscales AND Discussion, Paragraph 5 for scoring and response options |
| **6.** Provide a clear description of existing evidence on the quality of the PROM. | **Existing evidence on the quality of the PROM clearly described.** |  | Existing evidence on the quality of the PROM not clearly described. |  | Novel supplement for SCNS-SF34 – base measure quality in Discussion, Strengths & Limitations |
| **7.** Provide a clear description of the context of use.* | **Context of use clearly described.** |  | Context of use not clearly described. |  | Methods, Phase 4, Paper-administered AND Discussion, Implications, Usable in research and clinical contexts |
| ***Target population*** | | | | | |
| **8.** Provide a clear description of in- and exclusion criteria to select patients, e.g. in terms of disease condition and characteristics like age, gender, language or country, and setting (e.g. general population, primary care or hospital/rehabilitation care). | **Inclusion and exclusion criteria for patients clearly described.** |  |  | Inclusion and exclusion criteria for patients not clearly described. | Methods, Phase 2 |
| **9.** Provide a clear description of the method used to select the patients for the study (e.g. convenience, consecutive, or random). | **Method for patient selection clearly described.** |  | Method of patient selection not clearly described. |  | Methods, Phase 2, Purposive sampling |
| **10.** Describe whether the selected sample is representing the target population in which the PROM will be used in terms of age, gender, important disease characteristics (e.g. severity, status, duration). | **Study sample representing the target population clearly described.** | Assumable that the study sample is representing the target population, but not clearly described. | Unclear whether the study sample is representing the target population. | Study was not performed in a sample representing the target population. | Methods, Phase 2, Advanced cancer patients using IBP therapies |
| **Content validity** | | | | | |
| ***Design requirements*** | | | | | |
| **1.** From the perspective of the patients: use an appropriate method for assessing (1) the relevance of each item for the patients’ experience with the condition, AND (2) the comprehensiveness of the PROM, AND (3) the comprehensibility of the PROM instructions, items, response options, and recall period. | **Widely recognised or well justified method for qualitative research was used to assess the three aspects.** | Only quantitative (survey) method(s) were used or assumable that the method used was appropriate but not clearly described, but all three aspects were be assessed. | Not clear if patients were asked whether each item is relevant AND comprehensible AND whether items together are comprehensive, or doubtful whether the method was appropriate. | Method used was not appropriate or patients were not asked about the relevance, comprehensiveness or comprehensibility of all items. | Methods, Phase 3, Step 3, Cognitive interviews |
| **2.** From the perspective of professionals: use an appropriate method for assessing (1) the relevance of each item for the construct of interest, AND (2) the comprehensiveness of the PROM. | **Widely recognised or well justified method for qualitative research was used to assess the two aspects.** | Only quantitative (survey) method(s) were used or assumable that the method used was appropriate but not clearly described, but both aspects were assessed. | Not clear if professionals were asked whether each item is relevant AND items together are comprehensive or doubtful whether the method was appropriate. | Method used was not appropriate or professionals were not asked about the relevance or comprehensiveness of all items. | Methods, Phase 3, Step 3, Group workshops |
| **3.** Include professionals from all relevant disciplines. | **Professionals from all required disciplines were included.** | Assumable that professionals from all required disciplines were included, but not clearly described. | Doubtful whether professionals from all required disciplines were included or relevant professionals were not included. |  | Methods, Phase 3, Step 3 AND Results, Phase 3, Step 3 |
| **4.** Evaluate each item in an appropriate number of patients or professionals.  For qualitative studies.  For quantitative (survey) studies. | **≥7**  ≥50 | 4-6  30-49 | <4 or not clear  <30 or not clear |  | Results, Phase 3, Step 3, n=16 patients, n=8 professionals |
| **5.** Use skilled group moderators or interviewers. | **Skilled group moderators or interviewers were used.** | Group moderators or interviewers had limited experience or were trained specifically for the study. | Not clear if group moderators or interviewers were trained group moderators/interviewers were not trained and had no experience. |  | Methods, Phase 3, Step 3 |
| **6.** Base the group meetings or interviews on an appropriate topic or interview guide. | **Appropriate topic or interview guide was used.** | Assumable that the topic or interview guide was appropriate, but not clearly described. | Not clear if a topic guide was used or doubtful if topic or interview guide was appropriate or no guide used. |  | Methods, Phase 3, Step 3 |
| **7.** Record and transcribe verbatim the group meetings or interviews. | **All group meetings or interviews were recorded and transcribed verbatim.** | Assumable that all group meetings or interviews were recorded and transcribed verbatim, but not clearly described. | Not clear if all group meetings or interviews were recorded and transcribed verbatim or recordings were not transcribed verbatim or only notes were made during the group meetings/interviews. | No recording and no notes. | Methods, Phase 3, Step 3 |
| ***Analyses*** | | | | | |
| **8.** Use an appropriate approach to analyse the data. | **A widely recognised or well justified approach was used.** | Assumable that the approach was appropriate, but not clearly described. | Not clear what approach was used or doubtful whether the approach was appropriate. | Approach not appropriate. | Methods, Phase 3, Step 3 |
| **9.** Involve at least two researchers in the analysis. | **At least two researchers were involved in the analysis.** | Assumable that at least two researchers were involved in the analysis, but not clearly described. | Not clear if two researchers were included in the analysis or only one researcher was involved in the analysis. |  | Methods, Phase 3, Step 3 |
| **Measurement error and Reliability** | | | | | |
| ***Design requirements*** | | | | | |
| **1.** Use at least two measurements. | **At least two measurements.** |  |  | Only one measurement. | Methods, Phase 4, Administered twice |
| **2.** Ensure that the administrations will be independent. | **Independent measurements.** | Assumable that the measurements were independent. | Doubtful whether the measurements were independent. | Measurements NOT independent. | Methods, Phase 4 |
| **3.** Ensure that the patients will be stable in the interim period on the construct to be measured. | **Patients were stable (evidence provided).** | Assumable that patients were stable. | Unclear if patients were stable. | Patients were NOT stable. | Methods, Phase 4 AND Discussion, Strengths & Limitations |
| **4.** Use an appropriate time interval between the two measurements, which is long enough to prevent recall, and short enough to ensure that patients remain stable. | **Time interval appropriate.** |  | Doubtful whether time interval was appropriate or time interval was not stated. | Time interval NOT appropriate. | Methods, Phase 4, Two-week time interval |
| **5.** Ensure that the test conditions will be similar for the measurements (e.g. type of administration, environment, instructions). | **Test conditions were similar (evidence provided).** | Assumable that test conditions were similar. | Unclear if test conditions were similar. | Test conditions were NOT similar. | Methods, Phase 4 |
| **6.** Perform the analysis in a sample with an appropriate number of patients (taking into account expected number of missing values). | ≥100 patients | 50-99 patients | 30-49 patients | **<30 patients** | Results, Phase 4, n=27 completed measure at both administrations |
| ***Statistical methods for measurement error*** | | | | | |
| **7.** For continuous scores: calculate the Standard Error of Measurement (SEM), Smallest Detectable Change (SDC) or Limits of Agreement (LoA). | SEM, SDC, or LoA was calculated, and model or formula is clearly described. | SEM or SDC were calculated, but model or formula of the SEM or SDC is not described or not optimal. |  | SEM was calculated based on Cronbach’s alpha, or on standard deviation from another population. | ***Not applicable*** |
| **8.** For dichotomous/nominal/ordinal scores: calculate the percentage (positive and negative) agreement. | % positive and negative agreement was calculated. | **% agreement was calculated.** |  | % agreement was not calculated. | Methods, Phase 4 AND Results, Phase 4 |
| **9.** Provide a clear description of how missing items will be handled. | **The way missing items were handled is clearly described.** |  | The way missing items were handled is not clearly described. |  | Methods, Phase 4 AND Results, Phase 4 |
| ***Statistical methods for reliability*** | | | | | |
| **7.** For continuous scores: calculate an intraclass correlation coefficient (ICC). | ICC was calculated and model or formula of the ICC is clearly described. | ICC was calculated but model or formula of the ICC is not described or not optimal. | Pearson or Spearman correlation coefficient was calculated. | No ICC or Pearson or Spearman correlations calculated. | ***Not applicable*** |
| **8.** For dichotomous/nominal/ordinal scores: calculate kappa. | **Kappa was calculated.** |  |  | No Kappa was calculated. | Methods, Phase 4 |
| **9.** For ordinal scores: calculate a weighted kappa. | **Weighted Kappa calculated and weighting scheme is described.** |  | Unweighted Kappa was calculated or not described if Kappa was weighted. |  | Methods, Phase 4, Cohen’s (linear) weighted Kappa |
| **10.** Provide a clear description of how missing items will be handled. | **The way missing items were handled is clearly described.** |  | The way missing items were handled is not clearly described. |  | Methods, Phase 4 AND Results, Phase 4 |
| ^1^This is a shortened version of the COSMIN study design checklist: Mokkink, L. B., Prinsen, C. A., Patrick, D. L., Alonso, J., Bouter, L. M., De Vet, H. C., & Terwee, C. B. (2019). COSMIN Study Design checklist for Patient-reported outcome measurement instruments. Amsterdam, The Netherlands, 2019, 1-32.  ^2^The purpose of this checklist is to provide transparency on whether all important issues were considered in this development study on the initial measurement properties of SCNS-TARGET. Here, we report our alignment with general COSMIN recommendations and those specific to the measurement properties examined in this paper, namely, content validity, reliability, and measurement error.  ^3^Note that we have amended the tense of the rating descriptions to reflect that we have reported a study that has been conducted, not a protocol.  ^4^To aid future COSMIN evaluation of SCNS-TARGET, we have highlighted in bold, the ratings given in the authors’ own COSMIN evaluation of the study, as well as where each requirement is reported in the paper.  *The context of use refers to the intended application of the PROM (e.g. for research or clinical practice), to a specific setting for which the PROM was developed (e.g. for use in a hospital or at home) or to a specific administration mode (e.g. paper or computer‐administered). If the PROM was developed for use across multiple contexts, this should be described. | | | | | |

**SUPPLEMENTARY TABLE 2. Definitions of eleven unmet needs domains^1^**

| Domain of unmet need | Definition |
| --- | --- |
| *Activities of daily living* | Needs relating to essential activities necessary for everyday functioning |
| *Autonomy* | Needs relating to an individual’s ability to do things for themselves |
| *Economic* | Needs relating to an individual’s finances and resources |
| *Health care* | Needs relating to the individual’s access to treatment and/or support services and their relationship with health professionals |
| *Information* | Needs for more information, knowledge or understanding about all aspects of the cancer journey |
| *Physical* | Needs relating to a symptom of diagnosis or side effect of treatment |
| *Psychological* | Needs relating to feelings and emotions surrounding the cancer journey |
| *Role* | Needs relating to an individual’s responsibility and the part they plat at home and society |
| *Social* | Needs concerning relationships with friends, family and partners |
| *Spiritual* | Needs that relate to finding meaning, purpose, and value in life, though not necessarily religious |
| *Sexual* | Needs relating to an individual’s intimate relationships and sexual function |

^1^ from Phase 1 systematic review (22)

**SUPPLEMENTARY TABLE 3. Instruments considered for adaptation^1^, decisions and reasons for selection/exclusion**

| **Instrument** | **Decision** | **Reason(s)** |
| --- | --- | --- |
| **SCNS-SF34 [1]** | Shortlisted & selected | - Target population is people with all types and stages of cancer - Full instrument openly available - Widely used internationally - Validated in multiple languages - Other groups have developed add-on modules - Covers multiple unmet needs domains - Includes questions on information needs (which are not present in other shortlisted instruments) and which emerged as important in concept elicitation (phase 2) - Original response options considered confusing by PPI group, but alternatives have previously been tested |
| **CSS-25 [2,3]** | Shortlisted, then excluded | - Target population is people with all types and stages of cancer - Fully instrument not openly available; confidentiality agreement required to obtain additional information - Covers multiple unmet needs domains - Items relate to whether individual wishes to receive support from health professionals; this help may not be available in a research study, potentially limiting usefulness of instrument in research - Items primarily focus on distress. |
| **eHNA [4]** | Shortlisted, then excluded | - Target population is people with all types and stages of cancer - Used in many UK centres as part of routine follow-up - Covers multiple unmet needs domains - Version which was validated no longer appears to be available or in use; version used in clinical practice does not appear to correspond with validated version - PPI representatives did not like: layout (too many questions per page), response options (confusing, meaning of numerical rating unclear); items (too much repetition) |
| **BCNAS-32 [5]** | Excluded | - Target population too specific (bladder cancer) |
| **CaNDI  [6]** | Excluded | - Narrow focus (psychological domain) |
| **CNAT [7,8]** | Excluded | - Not clear if English version has been validated - Interviewer administered |
| **CNQ-sf [9]** | Excluded | - Unable to contact authors to address queries regarding scoring system. |
| **NA-ACP [10]** | Excluded | - Considered too burdensome for patients (132 items) |
| **NA-ALCP [11]** | Excluded | - Target population too specific (lung cancer) |
| **Ndiok [12]** | Excluded | - Limited items relating to unmet needs - Interviewer administered |
| **NEQ [13,14,15]** | Excluded | - Target population too specific (hospitalised patients) |
| **PCNQ V2 [16,17]** | Excluded | - Target population too specific (prostate cancer) |
| **PCNA-EAV [18]** | Excluded | - Culturally specific (Saudi-Arabian population) - Interviewer administrated. |
| **PNAP [19]** | Excluded | - Not specifically for cancer patients (target population those undergoing palliative care) - Interviewer administered |
| **s/b PNPC [20]** | Excluded | - Considered too burdensome for patients (138 items) - Not clear if English version has been validated |
| **s/b PNPC -sv [21]** | Excluded | - Not clear if English version has been validated |
| **PNI [22]** | Excluded | - Narrow focus (psychological domain) |
| **SCNAT-IP [23,24]** | Excluded | - Culturally specific (indigenous Australian population) - Interviewer administered |
| **SCNS-LF59 [25]** | Excluded | - Superseded by SCNS-SF34 |
| **SCNS-ST9 [26,27]** | Excluded | - Considered too brief |
| **SPARC [28]** | Excluded | - Not specifically for cancer patients (target population those undergoing palliative care) - Interviewer administered |
| **SPEED [29]** | Excluded | - Target population too specific (emergency department patients) - Interviewer administrated. |
| **YYFcore03 [30]** | Excluded | - Target population to specific (newly diagnosed patients) |
| **3LNQ [31]** | Excluded | - Limited items relating to unmet needs |

^1^ identified from Phase 1 systematic review (22)

**References for instruments:**

[1] Boyes A, Girgis A, Lecathelinais C. Brief assessment of adult cancer patients' perceived needs: development and validation of the 34‐item Supportive Care Needs Survey (SCNS‐SF34). J Eval Clin Pract 2009; 15(4): 602-6.

[2] Miller MF. Mullins CD, Onukwugha E, Golant M, Buzaglo JS. Descriminatory power of a 25-item distress screening tool: a cross-sectional survey of 251 cancer survivors. Qual Life Res 2014; 23(10): 2855-2863.

[3] Buzaglo JS, Zaleta AK, McManus S, Golant M, Miller MF. CancerSupportSource®: validation of a revised multi-dimensional distress screening programe for cancer patients and survivors. Support Care Cancer 2020; 28(1): 55-64.

[4] Snowden A, Fleming M. Validation of the electronic holistic needs assessment. Springerplus. 2015;4(1):1

[5] Leung TM, Benn E, Galsky M, Latini DM, Goltz HH, Lee CT, et al. Examining the psychometric properties of the bladder cancer needs assessment survey. JOJ Urol Nephrol. 2017;4:555627.

[6] Lowery AE, Greenberg MA, Foster SL, Clark K, Casden DR, Loscalzo M, et al. Validation of a needs-based biopsychosocial distress instrument for cancer patients. Psychooncology. 2012;21(10):1099–106.

[7] Shim EJ, Park JH, Park JH. Comprehensive needs assessment tool in cancer (CNAT): the development and validation. Support Care Cancer. 2011;19(12):1957–68.

[8] Yang GM, Pang GS, Lee GL, Neo PS, Wong YY, Qu DL, et al. Validation of the comprehensive needs assessment tool in patients with advanced cancer. Indian J Palliat Care. 2019;25(3):374–8.

[9] Cossich T, Schofield P, McLachlan SA. Validation of the cancer needs questionnaire (CNQ) short-form version in an ambulatory cancer setting. Qual Life Res. 2004;13(7):1225–33.

[10] Rainbird KJ, Perkins JJ, Sanson-Fisher RW. The needs assessment for advanced cancer patients (NA-ACP): a measure of the perceived needs of patients with advanced, incurable cancer. A study of validity, reliability and acceptability. Psychooncology. 2005;14(4): 297–306.

[11] Schofield P, Gough K, Ugalde A, Dolling L, Aranda S, SansonFisher R. Validation of the needs assessment for advanced lung cancer patients (NA-ALCP). Psychooncology. 2012;21(4):451–5.

[12] Ndiok A, Ncama B. Assessment of palliative care needs of patients/ families living with cancer in a developing country. Scand J Caring Sci. 2018;32(3):1215–26.

[13] Tamburini M, Gangeri L, Brunelli C, Beltrami E, Boeri P, Borreani C, et al. Assessment of hospitalised cancer patients' needs by the needs evaluation questionnaire. Ann Oncol. 2000;11(1):31–8.

[14] Annunziata MA, Muzzatti B, Altoè G. A contribution to the validation of the needs evaluation questionnaire (NEQ): a study in the Italian context. Psychooncology. 2009;18(5):549–53.

[15] Bonacchi A, Miccinesi G, Galli S, Primi C, Chiesi F, Lippi D, et al. use of the needs evaluation questionnaire with cancer outpatients. Support Care Cancer. 2016;24(8):3507–15.

[16] Duke JM, Treloar CJ, Byles JE. Evaluation of a revised instrument to assess the needs of men diagnosed with prostate cancer. Support Care Cancer. 2005;13(11):895–903.

[17] Duke JM, Treloar CJ, Byles JE. Evaluation of an instrument to assess the needs of men diagnosed with prostate carcinoma: an assessment of the validity and reliability of a self-administered questionnaire developed to measure the needs experienced by men diagnosed with prostate carcinoma. Cancer. 2003;97(4):993– 1001.

[18] Volker SE. Development and validation of a palliative care needs assessment instrument-English/Arabic versions (PCNA-EAV), for use with patients with advanced cancer: The University of Alabama at Birmingham; 2010.

[19] Buzgova R, Kozakova R, Sikorova L, Zelenikova R, Jarosova D. Development and psychometric evaluation of patient needs assessment in palliative care (PNAP) instrument. Palliat Support Care. 2016;14(2):129–37.

[20] Osse BH, Vernooij MJ, Schadé E, Grol RP. Towards a new clinical tool for needs assessment in the palliative care of cancer patients: the PNPC instrument. J Pain Symptom Manag. 2004;28(4):329–41.

[21] Osse BH, Vernooij-Dassen MJ, Schadé E, Grol RP. A practical instrument to explore patients’ needs in palliative care: the problems and needs in palliative care questionnaire—short version. Palliat Med. 2007;21(5):391–9.

[22] McIllmurray MB, Thomas C, Francis B, Morris S, Soothill K, AlHamad A. The psychosocial needs of cancer patients: findings from an observational study. Eur J Cancer Care. 2001;10(4):261–9.

[23] Garvey G, Beesley VL, Janda M, Jacka C, Green AC, O’Rourke P, et al. The development of a supportive care needs assessment tool for Indigenous people with cancer. BMC Cancer. 2012;12(1):1.

[24] Garvey G, Beesley VL, Janda M, O'Rourke PK, He VY, Hawkes AL, et al. Psychometric properties of an Australian supportive care needs assessment tool for Indigenous patients with cancer. Cancer. 2015;121(17):3018–26.

[25] Bonevski B, Sanson-Fisher R, Girgis A, Burton L, Cook P, Boyes A. Supportive care review group. Evaluation of an instrument to assess the needs of patients with cancer. Cancer. 2000;88(1):217– 25.

[26] Boyes A, Girgis A, Lecathelinais C. Brief assessment of adult cancer patients’ perceived needs: development and validation of the 34-item supportive care needs survey (SCNS-SF34). J Eval Clin Pract. 2009;15(4):602–6.

[27] Girgis A, Stojanovski E, Boyes A, King M, Lecathelinais C. The next generation of the supportive care needs survey: a brief screening tool for administration in the clinical oncology setting. Psychooncology. 2012;21(8):827–35.

[28] Ahmedzai SH, Payne S, Bestall JC, Ahmed N, Dobson K, Clark D, Noble B. Improving access to specialist palliative care: developing a screening measure to assess the distress caused by advanced illness that may require referral to specialist palliative care. 2005.

[29] Richards CT, Gisondi MA, Chang CH, Courtney DM, Engel KG, Emanuel L, et al. Palliative care symptom assessment for patients with cancer in the emergency department: validation of the screen for palliative and end-of-life care needs in the emergency department instrument. J Palliat Med. 2011;14(6):757–64.

[30] Wong FL, Stiller T, Obenchain R, David C, Patel SK, Xie B, et al. Validation of a biopsychosocial distress screening tool,“you, your family and coh are a team”. Psychooncology. 2019;28(12):2396– 405.

[31] Johnsen AT, Petersen MA, Pedersen L, Groenvold M. Development and initial validation of the three-levels-of-needs questionnaire for self-assessment of palliative needs in patients with cancer. J Pain Symptom Manag. 2011;41(6):1025–39.
